# A Knee Rehabilitation Exercises Dataset for Postural Assessment using Wearable Devices

**DOI:** 10.1038/s41597-025-04963-4

**Published:** 2025-04-11

**Authors:** Panagiotis Kasnesis, Theodora Plavoukou, Amalia Contiero Syropoulou, Lazaros Toumanidis, George Georgoudis

**Affiliations:** 1 ThinGenious PC, Marousi, 15125 Greece; 2 University of West Attica, Department of Electrical and Electronic Engineering, Egaleo, 12241 Greece

**Keywords:** Health occupations, Research data

## Abstract

This article introduces the KneE-PAD, which is a dataset consisting of knee rehabilitation exercises performed by 31 patients suffering from knee pathologies. In particular, a total of 267 patients were monitored over a 6-month period where they were asked to perform in physiotherapy centers without any supervision 3 common lower limb rehabilitation exercises (squats, leg extension and walking). At each participant a set of 8 sEMG and IMU sensors were placed at important lower limb muscle groups. After curating and grouping the wrongly executed exercises, 2 common wrong variations for each exercise were identified in 31 participants, while a total of 2,086 files are available with each one having an approximate duration of 4.2s. The goal of KneE-PAD is to be used for training machine learning algorithms for automatic postural assessment using only wearable sensors (sEMG and IMU), which could become a vital part of a virtual coach to supervise the patients and provide useful feedback to them while executing their prescribed rehabilitation exercises remotely.

## Background & Summary

People affected by a motor impairment due to orthopedic anomalies (e.g., osteoarthritis) or lower limb injuries (e.g., anterior cruciate ligament) may undergo a surgical procedure, such as knee replacement or a ligament reconstruction surgery. In such case, after having face-to-face sessions in a hospital or a rehabilitation center, exercises are prescribed to them to continue their rehabilitation journey in their home for around 3 months and be reevaluated afterwards^1^. This part of the rehabilitation process is not usually supervised by a physiotherapist, thus, necessitating patients to remain consistent to achieve their benefits^2^, which becomes even more challenging when it involves the elderly^3^.

To this end, virtual assistants have been proposed to monitor and motivate the patients remotely through Virtual Reality (VR)^4^ or Extended Reality (XR)^5^ applications. Most of these rely on Machine Learning (ML)-based coaches capable of assessing the patient’s posture or 3D pose while executing a rehabilitation exercise^6^. In terms of data, several data sources have been proposed to capture the user’s movements and feed the ML algorithms. Vision-based approaches exploit RGB (Red, Green, and Blue) images^7^ and/or depth videos (RGB-D)^8–11^ to recognize the type of rehabilitation exercise and assess it. However, these technologies suffer from occlusions while not being privacy-aware. Thus, wearable-based approaches such as Inertial Measurement Units (IMUs)^12^, which capture the body kinematics, and surface Electromyography (sEMG)^13^, which capture the muscles activations, have been proposed as alternative solutions. Both of these modalities complement each other, since we can infer the muscle activations using inverse dynamics exploiting the IMUs^14^ or the body movements using inverse kinematics using sEMG data^15^. Nevertheless, these procedures involve complex modeling or ML-based techniques to approximate them while including both IMUs and sEMG which would directly measure them.

In addition to this, existing wearable-based datasets that include these two modalities are not designed for postural assessment^16–19^, thus, there was the need of building a dataset including rehabilitation exercises. Moreover, datasets collected to assess rehabilitation exercises, with the exception of FineRehab^20^, have been collected using healthy subjects instead of patients, asking them to perform in a wrong way the defined exercises. However, these data fail to accurately represent patient behaviors and irregular muscular activations and movements.

To the best of our knowledge, KneE-PAD (Knee Rehabilitation Exercises for Postural Assessment Dataset) is the first publicly available dataset that contains both sEMG and IMU data from patients performing rehabilitation exercises. In particular, it contains data from 31 patients suffering from knee pathologies that performed without any supervision 3 common lower limb rehabilitation exercises (squats, leg extension and walking), where 2 common wrong variations for each exercise were identified afterwards. KneE-PAD contains a total of 2,086 files with each one inducing a set of synchronized 8 sEMG and IMU sensors. The goal of KneE-PAD is to be used for training ML algorithms for the task of postural assessment using only wearable sensors signals. These algorithms could be integrated with AR/VR technologies to supervise, by providing useful feedback, and motivate the patients to execute their prescribed rehabilitation exercises.

## Methods

### Participants and ethical requirements

The dataset includes the lower limb rehabilitation exercises performed by 31 participants. The subject’s identities have being pseudo-anonymized using number IDs (i.e., not names, emails, etc). Table 1 presents their personal details, with M standing for masculine and F for feminine. Their age ranges from 18 to 68 years old, their height from 152 to 200 cm and their weight from 57 to 146 Kg. Moreover, for each participant we include the injured leg (left or right) and the corresponding pathology (e.g., osteoarthritis, meniscus, etc). This study was approved by the Research Ethics Committee of the University of West Attica (Approval No. 65417, 29/07/2023). It should be noted, also, that all participants signed a written informed consent for the collected data to be published.

**Table 1:** Anthropometric data, age, weight, height, sex, leg placement and knee pathology of volunteers.

Participant ID	Gender (M/F)	Height (cm)	Weight (kg)	Age (years)	Leg	Pathology
1	Male	182	87	27	left	meniscus
2	Male	182	118	47	left	meniscus
3	Female	152	83	56	left	osteoarthritis
4	Female	160	83	48	right	osteoarthritis
5	Male	181	85	31	right	tendonitis
6	Female	168	75	54	left	osteoarthritis
7	Female	162	90	63	right	osteoarthritis
8	Male	187	105	43	right	meniscus
9	Female	168	85	55	right	osteoarthritis
10	Female	164	94	51	right	meniscus
11	Male	197	108	49	right	cruciate ligament
12	Male	175	80	50	right	meniscus
13	Female	175	90	40	right	meniscus
14	Female	156	72	62	right	osteoarthritis
15	Male	177	84	38	right	meniscus
16	Female	175	95	55	left	ligament rupture
17	Female	172	72	58	left	osteoarthritis
18	Male	200	146	33	left	meniscus
19	Male	175	65	18	right	cruciate ligament
20	Female	157	84	51	left	osteoarthritis
21	Female	162	57	27	right	meniscus
22	Male	170	86	41	right	osteoarthritis
23	Female	164	85	51	right	meniscus
24	Female	169	82	51	left	osteoarthritis
25	Male	185	95	29	right	meniscus
26	Female	162	59	42	right	osteoarthritis
27	Female	166	75	68	left	osteoarthritis
28	Female	165	70	53	right	osteoarthritis
29	Male	182	84	41	right	meniscus
30	Male	175	90	47	left	osteoarthritis
31	Female	157	69	56	right	osteoarthritis

### Acquisition setup

For the creation of KneE-PAD we used 8 sEMG Delsys Trigno Avanti sensors^21^, which are equipped with IMU sensors also. In particular, they are very compact and lightweight with each one having a size of 27 x 37 x 13 mm and weighting 14 g, while their battery can last up to 8 hours. Each sensor records 1x EMG channel and 6x IMUs (i.e., 3-axial accelerometer and gyroscope). Moreover, we configured the sEMG sensor at 1259.259 Hz as sampling rate and that the IMU sensor at 148.148 Hz. The sEMG range is 11 millivolt (mV) having a bandwidth of 20-450 Hz, the accelerometer range is  ± 2 g at a 24-470 Hz bandwidth and the gyroscope’s range is 250 deg/s with a bandwidth of 24-360 Hz.

**Fig. 1:**
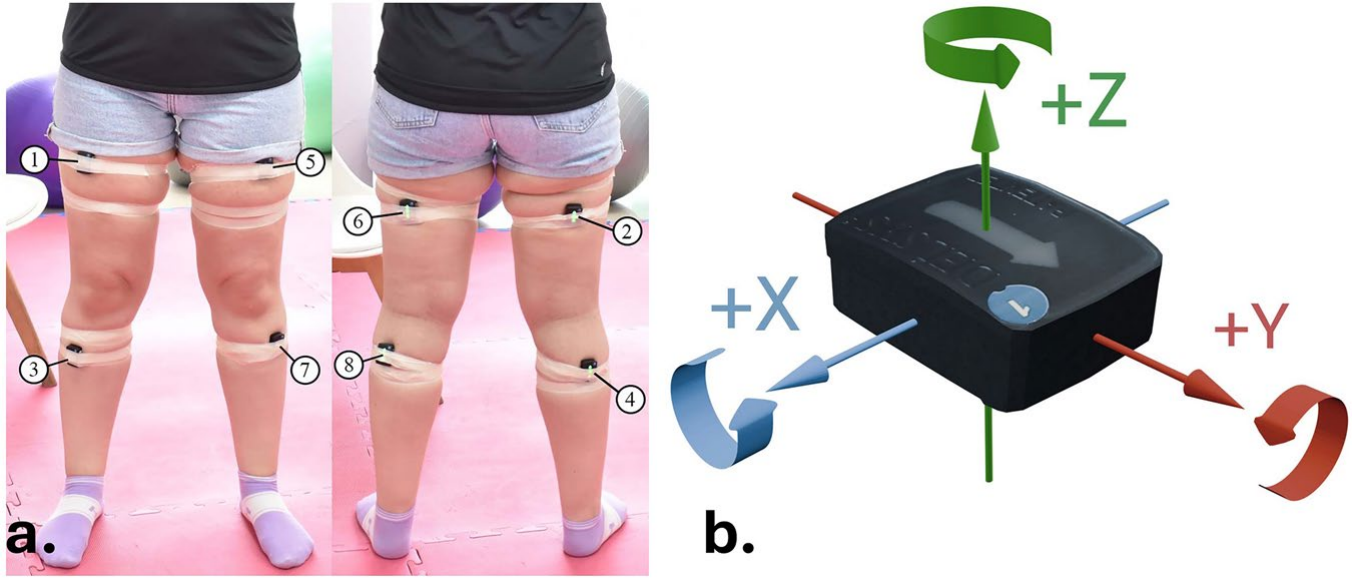
Illustration of the sensor placement including their ID (**a**) and their X, Y, Z orientation (**b**).

Figure 1a displays the placement of the sensors along with their corresponding ID (see Table 2). In particular, 4 sensors were placed at each leg at the following lower limb muscles: a) rectus femoris, b) hamstrings, c) tibialis anterior and d) grastocnemious. In addition to this, Fig. 1b shows the X, Y, Z-axis orientation of the selected sensors. Z-axis (roll axis) measures the acceleration of on the horizontal plane (forward and backward movements), while X (pitch axis) and Y-axis (yaw axis) on the vertical, with the former measuring the side acceleration (left or right) and the latter the height acceleration.

**Table 2:** Sensors’ placements on muscles.

Sensor ID	Muscle
1	right rectus femoris
2	right hamstrings
3	right tibialis anterior
4	right gastrocnemius
5	left rectus femoris
6	left hamstrings
7	left tibialis anterior
8	left gastrocnemius

Finally, all the participants wore a heart rate sensor, a muscle oxygenation sensor to monitor their level of discomfort, a goniometer to track the angle of the injured knee, while an RGB camera was used to record their sessions. It should be noted that these data were used for offline annotation purposes and are not provided within KneE-PAD. All the recorded modalities, apart from the camera output, were captured and synchronized using the Trigno Discover software^22^.

### Acquisition protocol

Over a 6-month period, 267 patients were observed while they independently performed three common lower limb rehabilitation exercises, wearing the selected set of 8 sEMG and IMU sensors. The data collection process took place at 2 rehabilitation centers where the patients executed their rehabilitation routine without any supervision from the physiotherapists to emulate an out-of-rehabilitation-center environment. The selection of the rehabilitation exercises was made according to the biomechanical kinematics of the knee joint. More specifically, the knee joint performs the main movements of flexion and extension combined with a small degree of rotation, and these movements can be carried out in both closed and open kinetic chains^23^. Thus, it was decided to perform flexion and extension in an open kinetic chain, with participants extending and bending the knee from a seated position (i.e., seated leg extension), and to perform flexion-extension in a closed kinetic chain by performing squats. This standardized program was structured following international guidelines as suggested by the literature^24–26^. Moreover, we included gait as a third rehabilitation exercise following the *Timed Up and Go* (TUG) test protocol, which was introduced in 1991 by Podsiadlo and Richardson^27^ as a measure of physical performance. Here, the patient is asked to rise from a chair 45-50 cm in height without armrests, come to a standing position, walk a distance of 3 m as quickly as possible, turn around and return to his/her original seated position. The total time of the test is related to the patient’s level of functional ability. In addition to this, it is worth noticing, that each patient was asked to repeat each rehabilitation exercise for 10 times (e.g., trials).

Using the recorded wearable-based data, along with the visual recordings from the RGB camera and the knee angles from the goniometer we curated and grouped the wrongly executed exercises in 2 common wrong variations for each exercise, with at least one identified in 31 participants that constitute the published dataset. The correct exercises along with their wrong variations are described in Table 3, providing an acronym and the corresponding “Class ID” (for machine learning purposes).

**Table 3:** Description of exercises and corresponding class IDs.

Exercise	Description	Execution Type	Class ID
*Squat*	From standing position, participants make the exercise sitting on chair, avoiding the lateral bending of knees or hip, and when touching the chair they stand up again.	Correct	0
*Squat_WT*	Squat with weight transfer on the healthy leg.	Wrong	1
*Squat_FL*	Squat by placing the injured leg in front.	Wrong	2
*Extension*	Sat on a chair, from the initial position of 90^∘^ of the knee flexion keeping the healthy leg still the injured one moves in the sagittal plane extending the knee until its maximum.	Correct	3
*Extension_NF*	Extension of the injured knee without full range motion.	Wrong	4
*Extension_LL*	Extension of the injured knee with lifting of the limb from the chair.	Wrong	5
*Gait*	Participants are asked to rise from a chair with a height of 45-50cm without armrests, come to a standing position, walk freely for a distance of 3 meters, turn and return to the standing position.	Correct	6
*Gait_NF*	Walking with the injured limb not full knee extension	Wrong	7
*Gait_HA*	Walking with the injured limb in full knee extension with hip abduction	Wrong	8

**Fig. 2:**
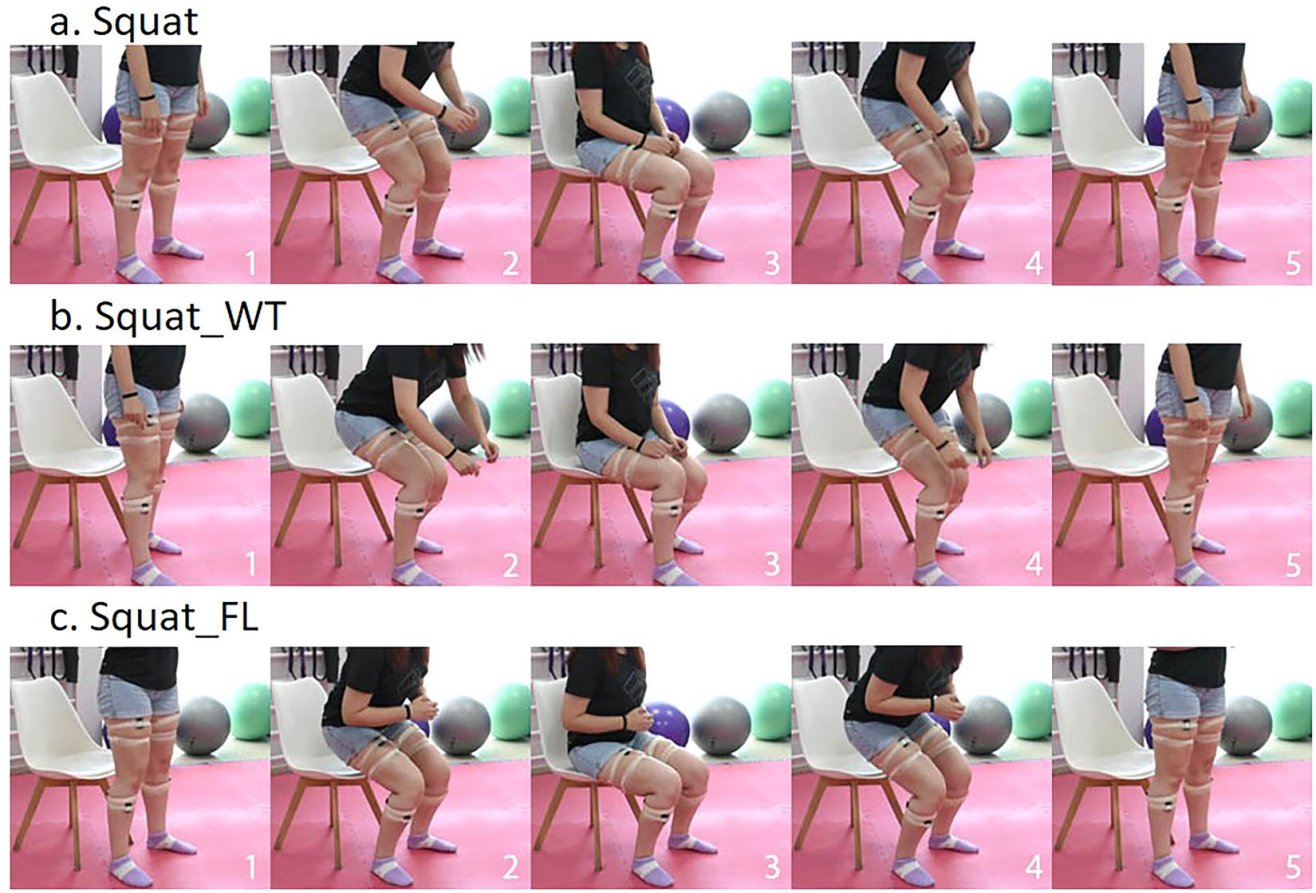
Visual example illustration of the performed squat variations: (**a**) *Squat*, (**b**) *Squat_WT*, and (**c**) *Squat_FL*.

In particular, for the case of the correct squat execution the participants started from the standing position and when they touched a chair placed behind them they stand up again (Fig. 2a). The first wrong variation is the *Squat_WT*, where there was a significant weight transfer on the healthy leg (Fig. 2b) and the second is the *Squat_FL*, where the participant wrongly placed the injured leg in front of the healthy one (Fig. 2c).

**Fig. 3:**
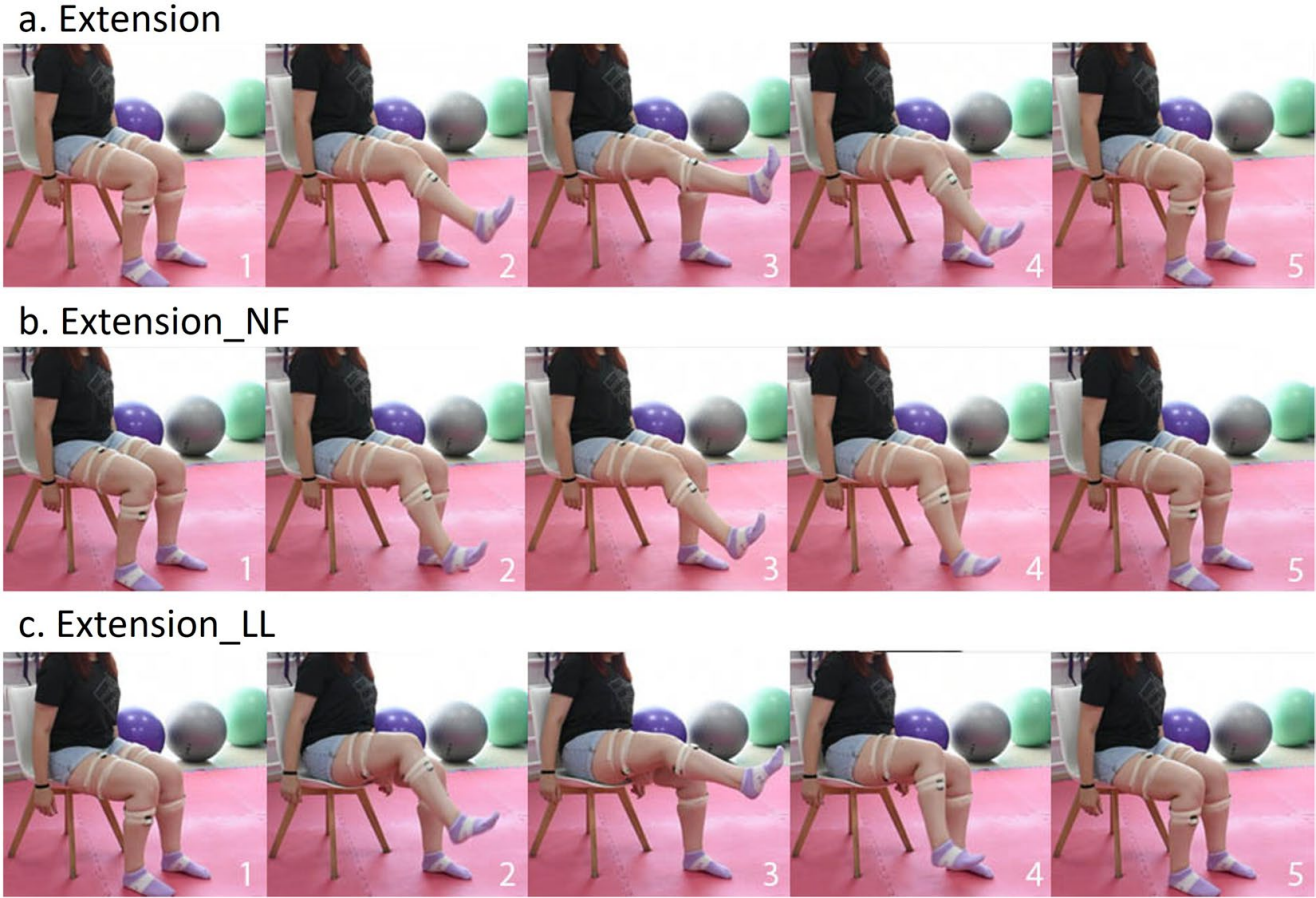
Visual example illustration of the performed seated leg extension variations: (**a**) *Extension*, (**b**) *Extension_NF*, and (**c**) *Extension_LL*.

The thee variations of the seated leg extension exercise are shown in Fig. 3. In Fig. 3b3 it is clear that the subject does not fully extend the knee, while Fig. 3c3 shows that the subject has lifted and abducted her leg in order to reach an almost full extension.

**Fig. 4:**
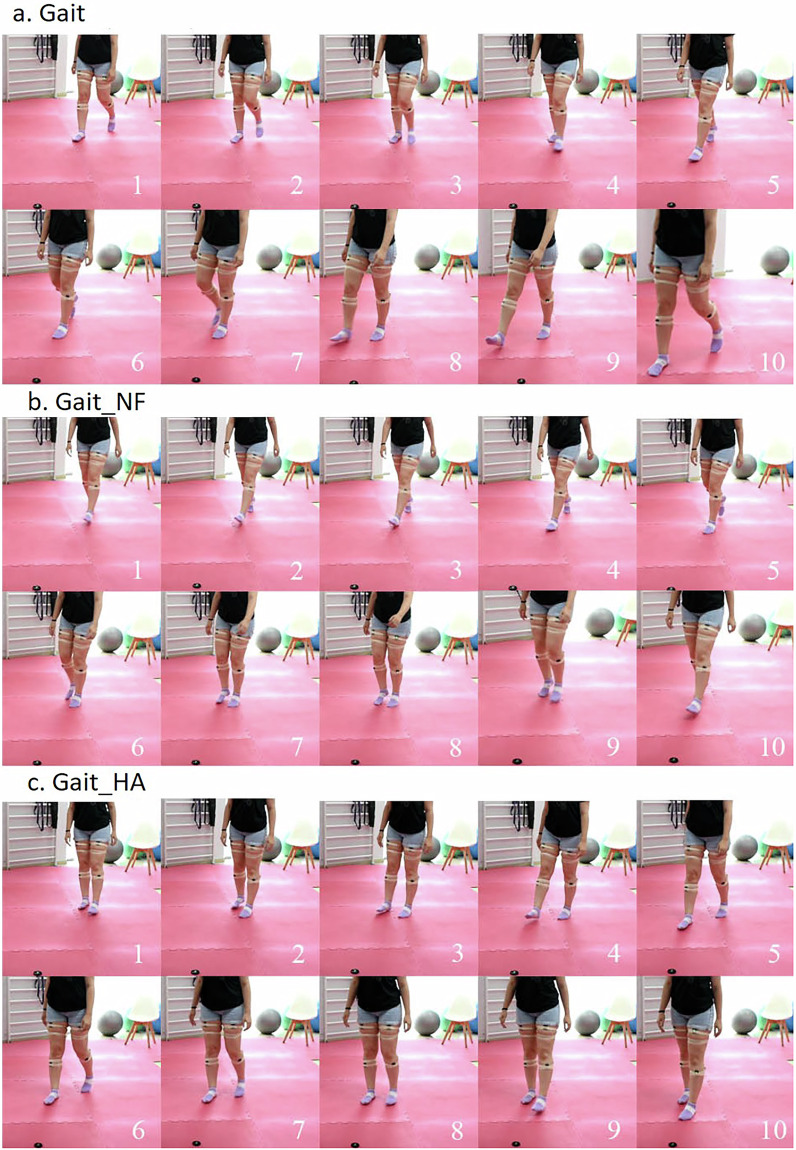
Visual example illustration of the performed gait variations: (**a**) *Gait*, (**b**) *Gait_NF*, and (**c**) *Gait_HA*.

Finally, Fig. 4 displays a full gait cycle of the gait variations from a subject having a knee pathology on the right leg. In Fig. 4b6-10 it is obvious that the subject during the *Gait_NF* does not fully extend the injured leg, placing almost next to the healthy one when touching the ground instead of forward. On the other hand, Fig. 4c3-8 shows that for the case of the *Gait_HA* variation the subject barely flexes her leg and abducts in order to move forward.

## Data Records

The raw data files exported from the Trigno Avanti sensors were stored as *.shpf* files, which is a proprietary file format provided by Delsys. To this end, for reusability purposes we converted all the files in *.npy* format. Moreover, we discarded unnecessary information included (e.g., sampling rate) keeping only the important values (sEMG and IMU data) and uploaded them to Zenodo^28^. In particular, a total of 2,086 files are available, with each one having an approximate duration of 4.2s and containing around 87.75M sEMG and 61.94M IMU samples.

**Fig. 5:**
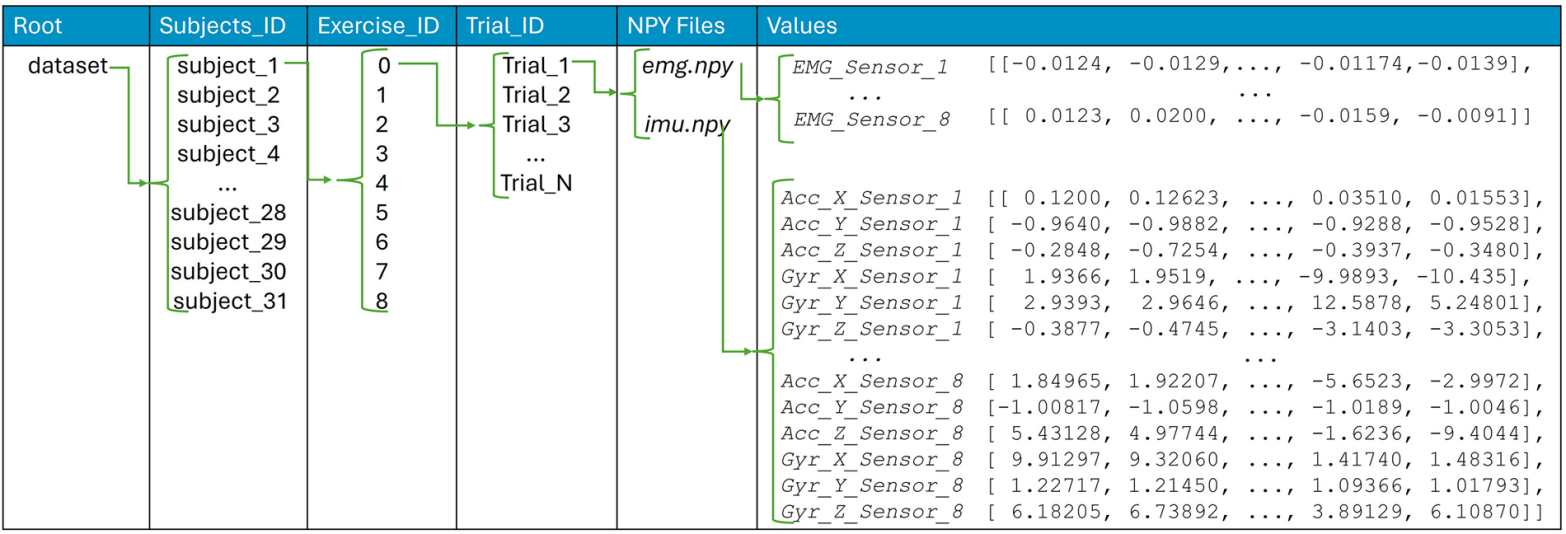
Data organization of KneE-PAD. The “Subject_ID” folders are included in the root directory, “dataset”. The subject-dependent folders are slit into activity classes ranging from 0 to 8, which are further divided into the executed repetitions (“Trial_ID”). Each of these contain the recorded sEMG (*emg.npy*) and the IMU samples (*imu.npy*).

The root folder “dataset” is divided in 31 subfolders (i.e., one folder for each participant), using the pseudo anonymization IDs shown in Table 1. Every subject folder (e.g., “Subject_3”) consists of the activities subfolders named using the “Class ID” depicted in Table 3 (e.g., “0”). It should be noted that some of the activities are missing since not all of the participants executed them correctly or in the way we categorized the poorly performed activities. The trials (i.e., repetitions) are included inside each activity-related folder, which may also vary for each activity. Finally, each trial forder contains an *emg.npy* and an *imu.npy* file, where the sEMG and IMU samples have been recorded, respectively. The tree-structure of the KneE-PAD is illustrated in Fig. 5.

### Data description

The provided *.npy* files contain the values obtained by the 8 Trigno Avanti sensors. Each sEMG file is comprised of 2D array, with the rows representing the “Sensor ID” (see Table 2) in ascending order and the rows the samples recorded for the particular session. For example, a 4-second session leads to an 8 × 5037 matrix containing sEMG values. Similarly, each IMU file consists of 2D array. Since IMUs produce 6 channels instead of 1 that the sEMG produces, here the order of the modalities is the following. The first 6 rows contain the samples recorded by the IMU sensor with ID equal to 1 (i.e, placed in the right rectus femoris muscle) and more specifically, the accelerometer sensor values (X, Y, Z-axis) are placed first, with the 3-axial gyroscope sensor values placed afterwards. The same pattern continues for the other IMU placements with the last 6 values obtained by “Sensor_8” (Fig. 5). For the IMU case, a 4-second session leads to a 48 × 593 matrix.

### Processed data

The sEMG signals are known to be vulnerable to movement artifacts and baseline noise^29^. To this end, we applied High-Pass Filtering (HPF) which is a fundamental signal processing to remove the low-frequency spectra of the noise signals that overlap with that of the sEMG signal^30^. In particular, we empirically used a high-pass filter (Butterworth 2^*nd*^ order) with a 50Hz cut-off (i.e., all frequencies below the cutoff are attenuated to zero). Moreover, we applied clipping at 0.3 mV since the studied muscles of the participants produced sEMG activities having less than this value, with larger values being considered as artifacts.

The enhanced signals have been then segmented into 4-second windows. This process is common when it comes to time-series analysis using machine learning algorithms and more specifically deep learning algorithms (e.g., Convolutional Neural Networks) that demand all the input data to have the same shape. It is worth mentioning that we selected for the rolling window a time step equal to 250 ms for the squat and extension activities while a 500 ms step was used for the gait activities. This choice was made to augment more the static activities and less the walking ones, since their duration of the latter is larger. The segmentation led to creation of 4,833 examples (i.e., a) *Squat*: 840, b) *Squat_WT*: 475, c) *Squat_FL*: 432, d) *Extension*: 404, e) *Extension_NF*: 318, f) *Extension_LL*: 480, g) *Gait*: 485, h) *Gait_NF*: 704, and i) *Gait_HA*: 695), each one having 8 × 5037 sEMG samples and 48 × 593 IMU samples.

**Fig. 6:**
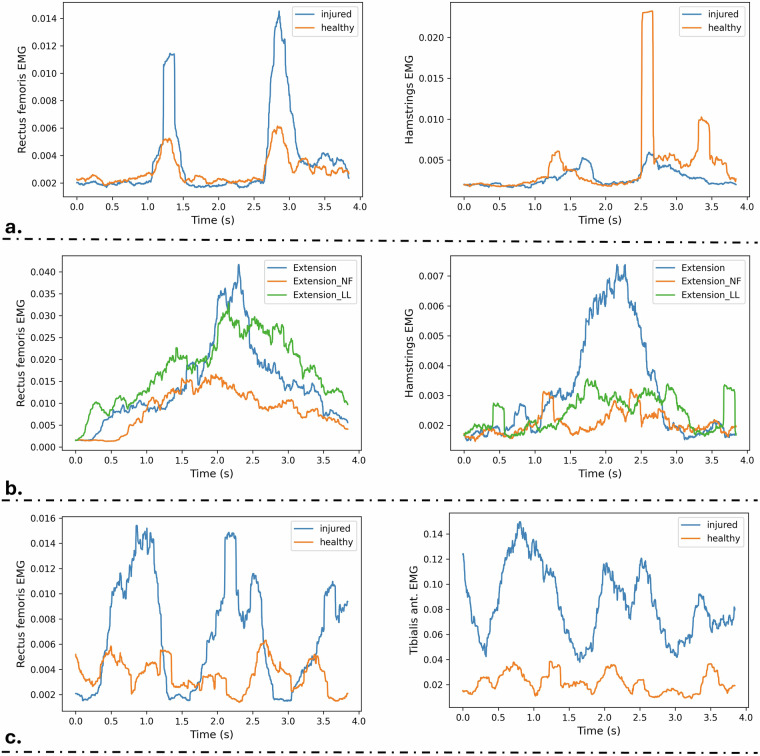
Processed signal examples. The upper part (**a**) includes the squat variations, the middle part (**b**) the variations of seated leg extensions and the lower part (**c**) the gait variations.

Some indicative examples of the produced sEMG signals after applying a sliding root mean squared function, for visualization purposes (i.e., signal smoothing), is illustrated in Fig. 6. Figure 6a at left shows vividly the differences of the rectus femoris activations of the healthy and the injured leg of a participant performing *Squat_WT*, while at the right the hamstring differences are presented for a *Squat_FL* session. As expected, in the latter squat variation the injured leg is placed front of the healthy one putting to much pressure on its hamstring. Figure 6b depicts the activation differences of the rectus femoris (left) and hamstring (right) of the injured leg while performing the correct extension exercise and the two wrong variations. For the case of the rectus femoris a significant difference (over 100%) is presented when comparing its activation on performing the *Extension_NF* exercise with the *Extension* and the *Extension_LL*, which is expected since the leg is not fully extended. Similarly, the hamstring is also barely activated, for the case of the two wrong variations. Finally, *Extension_NF* presents the gait variations. At the left the *Gait_NF*-based rectus femoris activations are presented for the healthy and injured legs, with the latter being underactivated due to not full extension, and on the right the *Gait_HA*-based tibialis activations showcase due to the hip abduction this muscle is not as active as it should be.

### Metadata

The *metadata.txt* file contains information about: (i) the subjects (ID, age, weight, height, sex, leg placement and knee pathology), (ii) the sensor configuration for Delsys Trigno Avanti Sensors (frequency, number of channels, units), (iii) the sensors’ placements with respect to their ID, and (iv) the description and IDs of the monitored exercises.

## Technical Validation

### Exploratory data analysis

**Fig. 7:**
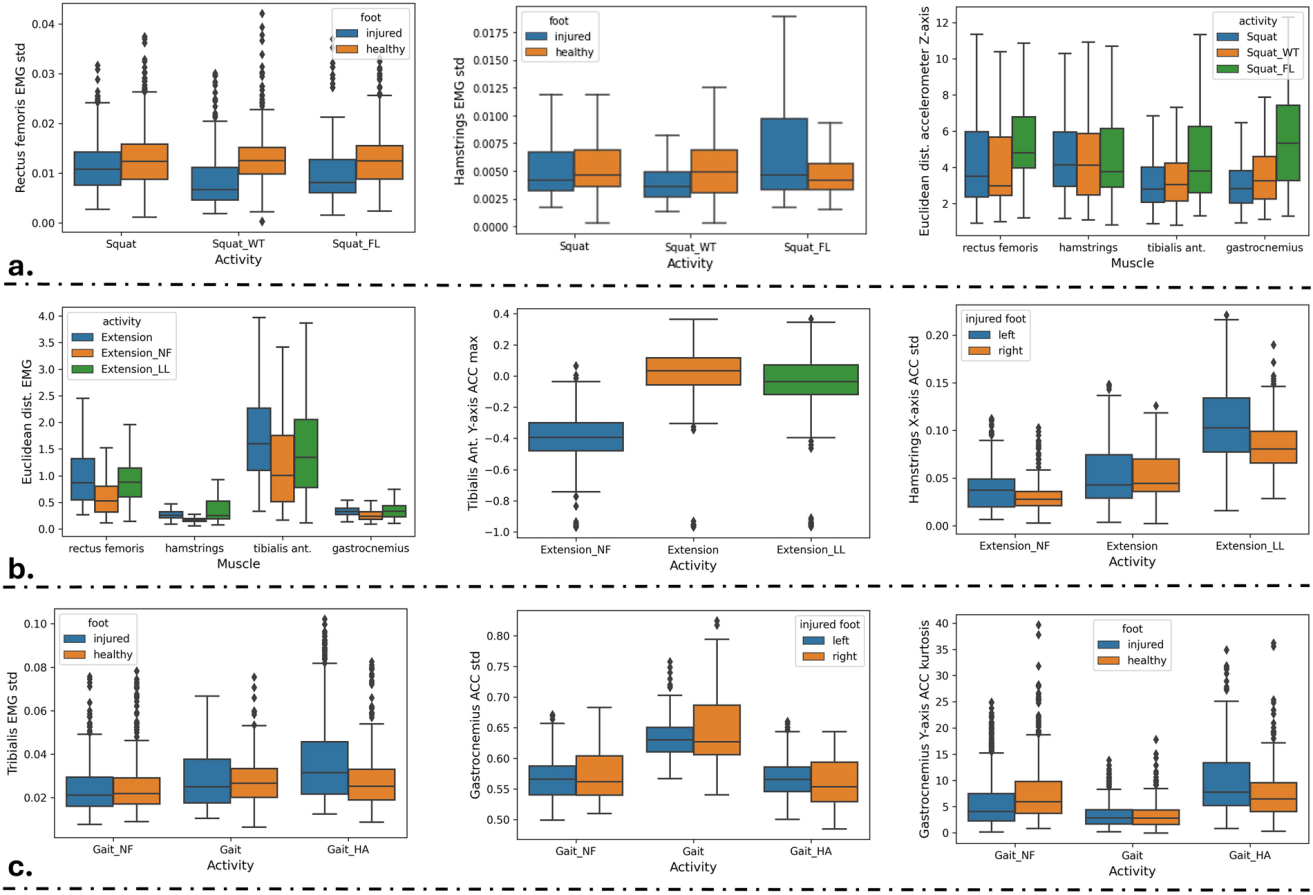
EDA performed on the squat variations (**a**), seated leg extension variations (**b**) and the gait variations (**c**).

The objective of this section is to validate the technical quality of the built dataset by performing an Exploratory Data Analysis (EDA). To this end, the extracted statistical properties (i.e., features) should have a bio-mechanical explanation, while taking into consideration that all the participants suffered from a knee pathology (i.e., not healthy). Thus, some variations when comparing the muscle activations or kinematics between the healthy and injured leg, even in the correctly performed exercises, are expected. The main characteristics of the performed EDA are summarized using statistical graphics in Fig. 7, where the upper part (a) includes the variations of squat, the middle part (b) the variations of seated leg extension and the lower part (c) the gait variations.

In Fig. 7a at the left the standard deviation *σ* of the rectus femoris activation is depicted, where the difference between the healthy and the injured leg for the *Squat_WT* is much higher compared to the other squat variation, since the user’s weight is transferred on the healthy leg. Similarly, the *σ* of the hamstring activation is higher for the injured leg when performing *Squat_FL*. Finally, at the right of Fig. 7a we can see the estimated Euclidean distances between the obtained accelerations of the Z-axis (forward view of the user, see Fig. 1b) of the healthy and the injured legs for every sensor placement. The most significant difference is observed for the case of the sensor placed on the gastrocnemius, where the mean Euclidean distance estimated for the case of the *Squat_FL* variation is more than x2 larger than that of the *Squat* and more than x1.5 larger compared to the corresponding value of the *Squat_WT*. This is expected if we consider the fact that this body part along with the tibialis anterior, where the same pattern is also observed, is extended more in the Z-axis compared to the rectus femoris and hamstrings.

Moving to Fig. 7b at the left we observe the estimated Euclidean distances between the obtained sEMG signals of the healthy and the injured legs per sensor placement. For all the placements, the distances for the *Extension_NF* variation are smaller compared to the other two variations, meaning that, since the muscle activations of the healthy leg should be close to zero, the patient does not fully extend his/her leg leading to smaller activations. In the middle, the maximum tibialis anterior Y-axis accelerations are depicted for the case of the injured leg, where again the not fully extended leg leads to less movement of the tibialis towards the vertical axis. For the case of the *Extension_LL* variation the most significant statistical difference when compared to the correct extension is observed on the *σ* estimated for the X-axis acceleration (i.e., vertical side view) of the hamstring. This is expected from a biomechanical point of view since the patients in order to fully extend their legs, lifted their limb from the chair (Fig. 3b2), thus, even though the tibialis acceleration for these two exercises is almost identical, the performed leg abduction when executing the *Extension_LL* is captured by the X-axis accelerometer revealing the identity of the exercise.

At lower left part of the EDA illustration (Fig. 7c) the estimated *σ* of the tibialis sEMG signal is shown. As aforementioned, since the data are originated from people suffering from knee pathologies, even for the case of normal gait the *σ* values vary slightly between the injured and the healthy leg. The difference is more observable for the case of the *Gait_HA*, where the muscle activation of injured leg’s tibialis anterior is higher, since the patient keeps his foot flexed and consequently does not relax this muscle. Observing the plot at the lower middle, it is evident that the *σ* of the accelerations in all axes is much higher when the patient was walking normally, since in the wrong variations the patients tends to control its leg movement (flexion and/or extension) to avoid any potential pain. Finally, we estimated the kurtosis of the Y-axis acceleration of the gastrocnemius. Kurtosis generally indicates whether the distribution or the produced values is thin (high kurtosis values) or broad (small kurtosis values) and can be used as an indicator of normality, with the kurtosis of a normal distribution being equal to 3. Here, normal gait produces almost identical kurtosis values for the injured and the healthy leg, with the average value being almost close to 3. For the case of the *Gait_NF* variation the healthy leg’s kurtosis are higher than those of the injured, since during a gait cycle the patient tends to lower the injured leg and lift the healthy leg to support the not fully extended injured limb. The opposite pattern is shown by the *Gait_HA*, due to the abduction the patient performs on the injured limb (Fig. 4c3) leading to more sharp vertical movements (i.e., quick lift-offs).

### Comparison with public wearable-based datasets for postural assessment

In the current section, we encounter postural assessment datasets that are only publicly available (i.e., we exclude those that are not^13,31^). Although, wearable-based datasets including both IMUs and sEMG exit these are designed for human activity recognition^17–19^, and/or for lower limb joint’s angle estimation^11,16–18,32^ (useful to lower-limb prostheses and exoskeletons). They are not designed for postural assessment and they use healthy subjects in the experiments. To the best of our knowledge, KneE-PAD is the only publicly available dataset that is designed for rehabilitation assessment while containing both modalities and data from patients having knee pathologies.

Starting with unimodal datasets, PHYTMO^12^ includes the IMU recordings of 30 healthy volunteers performing a total amount of 6 rehabilitation exercises (upper and lower limb) and 3 gait variations. Each participant performed two sessions with a minimum of 8 repetitions in each one, producing 7,076 files in total. UI-PRMD^10^ is a vision-based dataset consisting of 10 rehabilitation movements, executed by 10 healthy individuals. Each movement was repeated 10 times (correct or incorrect) in front of two sensory systems for motion capturing an optical tracker, and an RGB-D camera, leading to 2,000 files. Thus, the data provides along with the exercise type, the body joints’ positions and angles. A completely different approach exploiting a millimeter-wave (mmWave) radar and an RGB-D camera as a reference (estimated joints’ angles and positions) is introduced by MARS^33^. The dataset contains the mmWave radar-produced 3D point cloud (including also the Doppler velocity and intensity) obtained by 4 healthy subjects performing 10 specific rehabilitation movements, while being supervised (no incorrect movements included).

When it comes to multi-modal dataset, FineRehab^20^ comprises 16 exercises executed by 50 participants. It is worth mentioning that this dataset contains both healthy individuals and patients with musculoskeletal disorders. The total dataset consists of 4,215 files captured by two Kinect (RGB-D) cameras and 17 IMUs, offering also the RGB-D based estimations of joints’ angles and positions. Similarly, Palermo *et al*.^34^ exploited the same sensor modalities to develop a dataset consisted of 14 healthy participants walking with a wheeled robotic walker. Consequently, this dataset is suitable for gait analysis, containing the 3D joints’ angles, since other rehabilitation exercises are not included.

### Limitations

After conducting the literature review presented in the previous subsection, we concluded that even though the collected dataset offers the advantage of including multi-modal patients data, it includes less sensor modalities compared to others^11,16– 18^. In particular, vision markers could be very useful in order to capture the ground truth joints’ angles of the patients, making the dataset also applicable for the development of 3D pose estimation algorithms. In addition to this, upper limb rehabilitation exercises are not included, reducing its application to knee rehabilitation exercises.

**Fig. 8:**
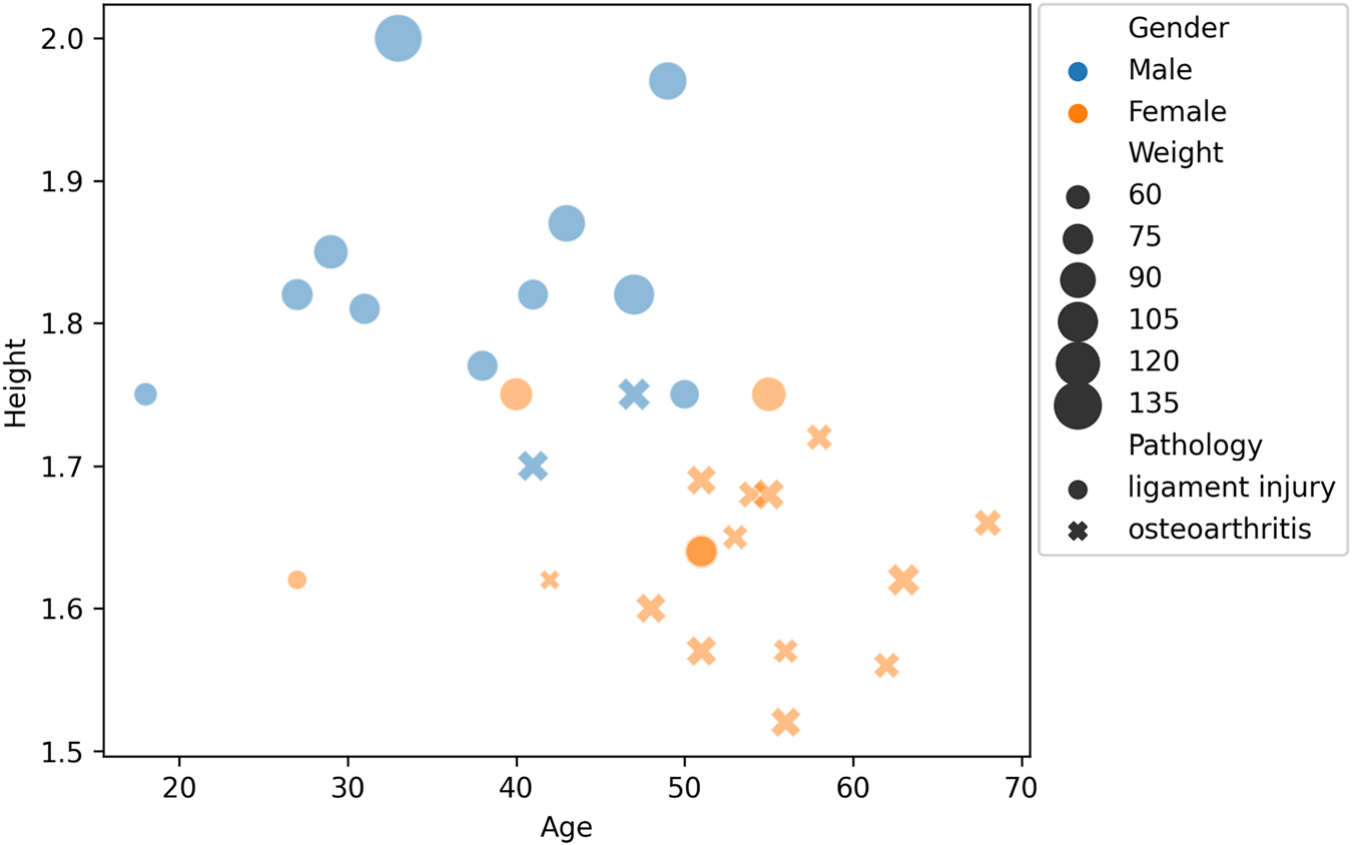
Patients’ demographics in terms of gender (blue or orange color), height (Y-axis), age (X-axis), pathology (“x” marker for osteoarthritis and “o” for ligament injuries), and weight (size of the marker).

Finally, there is a statistical bias on the patients’ data displayed vividly in Fig. 8. KneE-PAD is not highly imbalanced in terms of the participants gender, however, it is biased when it comes to the participant’s age and knee pathology. In particular, the included male patients are significantly younger than the female ones, while suffering a ligament injury, not osteoarthritis that female patients tend to do. Even though, this statistical bias is expected^35^, it limits the machine learning tasks the dataset could be used for. For example, for the case of knee pathology identification, although the gender and height could be left out of the feature set, the latter could be revealed by the range of patient movement of the sensor’s placement.

## Data Availability

The code for downloading, loading, pre-processing and applying the reported EDA can be found on GITHUB via the following URL (https://github.com/ounospanas/KneE-PAD). The code was written in Python programming language (3.8 version) and is provided in a *.ipynb* format (https://jupyter.org/). In particular, Numpy (https://numpy.org/) library was used for loading and pre-processing the data, scikit-image (https://scikit-image.org/) for data segmentation, Pandas (https://pandas.pydata.org/) along with Numpy for the statistical analysis, and Matplotlib (https://matplotlib.org/) and Seaborn (https://seaborn.pydata.org/) for the presented visualizations.
